# Tumor microenvironment characterization in cervical cancer identifies prognostic relevant gene signatures

**DOI:** 10.1371/journal.pone.0249374

**Published:** 2021-04-26

**Authors:** Linyu Peng, Gati Hayatullah, Haiyan Zhou, Shuzhen Chang, Liya Liu, Haifeng Qiu, Xiaoran Duan, Liping Han

**Affiliations:** 1 Department of Gynecology, The First Affiliated Hospital of Zhengzhou University, Zhengzhou, Henan, China; 2 Department of Occupational and Environmental Health, College of Public Health, Zhengzhou University, Zhengzhou, Henan, China; University of Calgary, CANADA

## Abstract

**Objective:**

The aim of this study is to systematically analyze the transcriptional sequencing data of cervical cancer (CC) to find an Tumor microenvironment (TME) prognostic marker to predict the survival of CC patients.

**Methods:**

The expression profiles and clinical follow-up information of CC were downloaded from the TCGA and GEO. The RNA-seq data of TCGA-CESC samples were used for CIBERSORT analysis to evaluate the penetration pattern of TME in 285 patients, and construct TMEscore. Other data sets were used to validate and evaluate TMEscore model. Further, survival analysis of TMEscore related DEGs was done to select prognosis genes. Functional enrichment and PPI networks analysis were performed on prognosis genes.

**Results:**

The TMEscore model has relatively good results in TCGA-CESC (*HR* = 2.47,95% *CI* = 1.49–4.11), TCGA-CESC HPV infection samples (*HR* = 2.13,95% *CI* = 1–4.51), GSE52903 (*HR* = 2.65, 95% *CI* = 1.06–6.6), GSE44001 (*HR* = 2.1, 95% *CI* = 0.99–4.43). Patients with high/low TMEscore have significant difference in prognosis (*log-rank test*, *P* = 0.00025), and the main difference between high TMEscore subtypes and low TMEscore subtypes is immune function-related pathways. Moreover, Kaplan-Meier survival curves found out a list of identified prognosis genes (n = 86) which interestingly show significant enrichment in immune-related functions. Finally, PPI network analysis shows that highly related nodes such as CD3D, CD3E, CD8A, CD27 in the module may become new targets of CC immunotherapy.

**Conclusions:**

TMEscore may become a new prognostic indicator predicting the survival of CC patients. The prognostic genes (n = 86) may help provide new strategies for tumor immunotherapy.

## Introduction

Cervical cancer(CC) is the fourth-ranked malignant tumor in female morbidity and mortality worldwide [1]. For the treatment of CC, surgical resection, chemotherapy, radiotherapy or comprehensive treatment methods are mainly used in clinical practice. Although the treatment effect for patients with early CC is relatively good, the treatment effect is poor for patients with persistent, advanced or recurring CC [2, 3]. Therefore, there is an urgent need for new biomarkers that can provide prognostic information to guide the prevention of CC metastasis and recurrence. In recent years, genome analysis has become the main method for discovering new biological targets in CC in the world [4, 5]. Interestingly, some studies have revealed the importance of tumor-related structures and the up-regulation of signaling pathways in cancer cells and the tumor microenvironment (TME) [6, 7], indicating that the intercellular relationships are more important than genomic factors at the single-cell level [8–10].

TME is a complex ecosystem consisting of various types of cells and their secreted products (such as cytokines, chemokines) and other non-cellular components of the extracellular matrix, with obvious heterogeneity, dynamics and complexity. The cell-to-cell correlation [11, 12]. TME plays a vital role in the growth and development of tumors [6, 13]. Tumor infiltrating immune cells (TICs) are an important part of TME, and there is a certain correlation between their infiltration patterns and clinical results. As we all know, CC is a malignant tumor that is highly related to human papillary virus (HPV) [14], considering the molecular mechanism of HPV-related CC immunotherapy provides another reasonable treatment option for CC [15]. In tumor immunotherapy, TICs play an important role in tumor control and response to treatment. Real-time understanding of the infiltration of immune cells in tumors is a very important indicator to guide clinical treatment [16, 17]. Therefore, analyzing the composition and characteristics of TICs in CC, as well as the correlation between the infiltrating pattern and prognosis, will help to better understand the complex anti-tumor response and guide effective immunotherapy in CC.

In the past, immunohistochemistry or flow cytometry was mostly used to analyze TICs in tumor tissues, which resulted in cumbersome procedures and low feedback efficiency. With the development of bioinformatics, many deconvolution methods can now be used to predict cell type and proportion information in complex tissue samples [17]. "Cell type Identification By Estimating Relative Subsets Of RNA Transcripts"(CIBERSORT) is a biology tool for bioinformation analysis based on linear support vector regression using the deconvolution method [19]. The CIBERSORT method can use standardized gene expression data to estimate the cell composition in different tumor samples. It has the advantages of high resolution and simultaneous quantification of multiple types of immune cells. Its superior performance has been verified in a variety of malignant tumors, such as colorectal cancer, breast cancer and lung adenocarcinoma [18–22]. Our study aims to use the CIBERSORT algorithm to analyze the gene expression data and clinical data of CC patients in TCGA to reveal the pattern of CC specific immune infiltration, and lay a foundation for revealing the potential biomarkers and targets of CC immunotherapy.

In our study, we estimated the TME infiltration pattern of TCGA-CESC CC patients for the first time, and obtained the TMEscore model through the principal component analysis algorithm. Importantly, by comparing the overall gene expression analysis and survival analysis of the TMEscore high/low groups, we obtained a list of prognostic genes (n = 86), which may help to describe the prognosis of patients with CC.

## Methods

### 1. Cervical cancer data sets and preprocessing

#### 1.1 Obtain gene expression profile data and clinical information of patients with CC from the TCGA database (https://tcga-data.nci.nih.gov/tcga/)

After removing duplicate samples and samples without survival information, there were 285 transcriptome samples used to verify TMEscore; further removing samples with survival time less than 30 days and samples without clinical information, there were 265 samples for differential analysis. Data preprocessing process:(a) Download CC RNA-seq count data from TCGA-CESC. (b) Delete adjacent samples. (c) Apply limma’s voom method to convert count data into CPM data, and then calculate weights according to the mean-variance relationship, so that the weighted data can be applied to linear models.

#### 1.2 Download the expression profile data and clinical data of 55/300 samples of GSE52903 and GSE44001 from GEO (https://www.ncbi.nlm.nih.gov/geo/) for verification

Both dataset of GEO data supported analysis, and neither sample were removed **(S1 Table)**.

### 2. Tumor microenvironment analysis

#### 2.1 Proportion of infiltrating cells in the tumor microenvironment

Using TCGA-CESC RNA-seq data from 285 CC samples for CIBERSORT (https://cibersort.stanford.edu/) analysis [18, 23], and scores of 22 immune cells were obtained using LM22 signature and 1000 permutation.

#### 2.2 Use unsupervised clustering to identify TME patterns and classify tumor samples into subgroups

According to the immune cell proportion data analyzed by CIBERSORT [18], the elbow (WSSE or within-cluster sum of squared error, this method is to find the best number of clusters by finding the "elbow point") and gap statics (The point at which Wk drops the fastest, K value corresponding to the maximum Gap) was used to evaluate the number K of the best category. The ConsensusClusterPlus R package [24] was used to classify to obtain TMEcluster (kmeans, euclidean, ward.D), and this procedure was repeated 1,000 times to ensure the stability of classification. Then we combined survival data to check whether this classification is related to survival.

#### 2.3.Calculate TMEscore and analyze whether TMEscore is related to survival

Based on the above TMEcluster results, map the clustering results to the RNA-seq data, and use limma R package [25] to screen differentially expressed genes (DEGs) for different TMEcluster types of samples. The screening threshold is *adj*.*P values* < 0.05 and | *log2FC* |> log2(1.5). Select class-specific differential genes, then use random forest classification algorithm to eliminate redundant genes to obtain signature genes [26], Next perform functional enrichment analysis on these genes to see which pathways are mainly enriched. The genes were divided into two categories (coefficient is positive or negative) using the cox regression model, and the TMEscore was calculated using the following formula with reference to the GGI score [27].

$$TMEscore=\sum log_{2}\left(X+1\right)‐\sum log_{2}\left(Y+1\right)$$

Equation 1: X is the expression value of the gene set whose Cox coefficient is positive, and Y is the expression value of the gene set whose Cox coefficient is negative.

Using the maximum selection test to find the best cut point, the samples were divided into two categories: TMEscore-High and TMEscore-Low, and the correlation between the two types of samples and prognosis was further analyzed.

#### 2.4 Use TCGA-CESC HPV infection samples, GSE52903 and GSE44001 data for validation

Based on the above results, TCGA-CESC HPV infection samples, GSE52903 and GSE44001 data were applied to the model to calculate TMEscore. Then the best pointcuts were found by the maximum selection test, which divides samples into TMEscore-high and TMEscore-low, and the correlation between the two samples and prognosis was analyzed. The single factor cox was used to calculate the 95% CI and HR of each factor in the forest diagram, HR is for TMEscore low vs TMEscore high.

### 3. Identification of differentially expressed genes

According to the grouping of the two types of TMEscore samples, limma package [25] was used to analyze the gene expression data. |log(Fold Change)|>1 and adj.*P* values<0.05 were set as the standards, and genes that meet the standards were defined as differentially expressed genes (DEGs).

### 4. Survival analysis

Survival analysis refers to the method of analyzing and inferring the survival time of organisms or people based on the data obtained from experiments or surveys, and studying the relationship between survival time and outcome and many influencing factors and their degree. It is also called survival rate analysis. DEGs were divided into high and low expression groups according to their median expression and subjected to survival analysis. The Kaplan-Meier diagram was drawn to illustrate the relationship between the overall survival (OS) of patients and the expression level of DEGs; Log-rank test was used to define DEGs with *P* <0.05 as prognostic genes related to survival.

### 5. PPI network construction

Prognosis genes related to survival were placed in a STRING database [28] (https://string-db.org/) to retrieve the protein-protein interaction (PPI) network and reconstructed via Cytoscape software [29]. At the same time, in order to identify the modules that are closely connected in the network, we used the Molecular COmplex DEtection (MCODE) plug-in (k-score = 3) and required the Degree Cut-off≥10 in the module to further mine the network, find the cluster according to the topology structure, and locate the densely connected modules.

### 6. Enrichment analysis

The clusterProfiler package [30] was used to identify and visualize the GO biological processes (BP) terms and KEGG pathways enriched by related genes. adj.*P* values < 0.05 was set as the cut-off criterion for the significant enrichment.

### 7. Statistical analysis

All Statistical analyses were conducted using R (https://www.r-project.org/), and *P* <0.05 were considered statistically significant. Unsupervised clustering methods: elbow method (R package factoextra), gap statistic (R package factoextra), consensus clustering (R package ConsensusClusterPlus). Differential expression analysis (R package limma). Correlation analysis (R function cor, pearson correlation). Maximum selection test to find the best cut-off point (R package maxstat). Cox regression (R package survival). The normality of the variables was tested by the Shapiro-Wilk normality test [31] For comparisons of more than two groups, Kruskal-Wallis tests was used as nonparametric methods [32]. The survival curve is generated by Kaplan-Meier method (R package survminer) and the difference is analyzed by log-rank test.

## Results

### 1. Tumor microenvironment analysis

#### 1.1 Infiltrating cells in the tumor microenvironment

**Fig 1:**
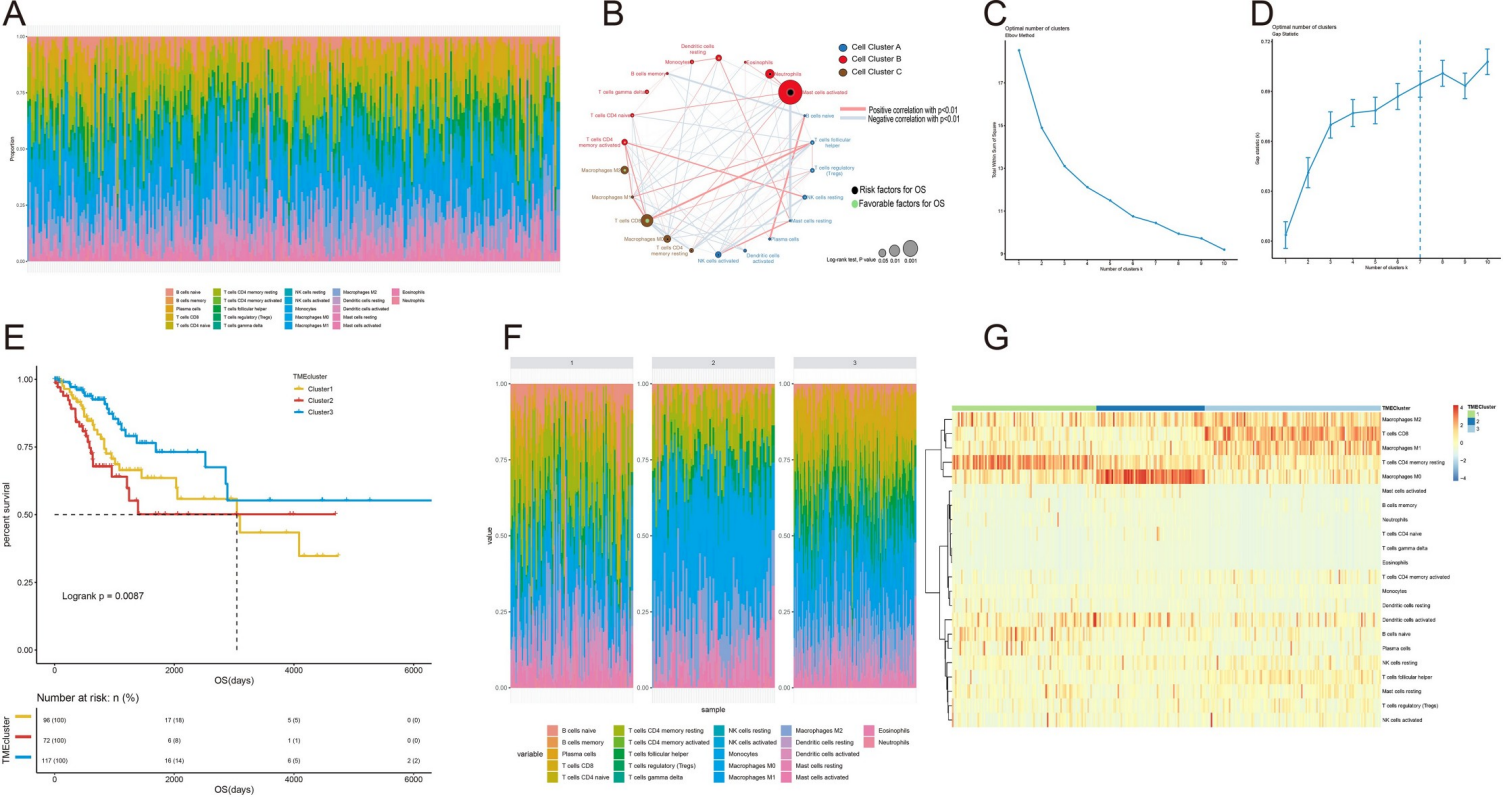
TME model classification. (A). The proportion of 22 types of immune cells in the sample. (B). 22 kinds of immune cells and their relationship with survival (The color of the dot represents the grouping, the size represents the relationship between survival. The color of the center point represents the prognostic risk. The connecting line between the points represents the correlation between the cell and the cell, the thickness of the line indicates the strength of cell correlation and the color of the line represents whether the correlation is positive or negative). (C)-(E) determine the optimal classification K = 3. (C). elbow method: vertical axis represents total within sum of square, horizontal axis represents number of clusters. (D). gap statics: vertical axis represents gap statistics, horizontal axis represents number of clusters. (E).Survival analysis of the 3 different TMEcluster. Kaplan–Meier curves for OS of 285 patients in the TCGA-CESC cohort showing the association between TMEcluster and OS (log-rank test, P< 0.001). (F). The proportion of immune cells in different TMEcluster. (G). Heat map of proportional clustering of immune cells in different TMEcluster.

CIBERSORT analysis [24] was performed using RNA-seq data from TCGA-CESC cohort to obtain the proportion of 22 immune cells (B cells memory, Dendritic cells activated, Macrophages M0, etc.) in 285 samples. As shown in **Fig 1A**, the proportion of immune cells in different samples is distributed; **Fig 1B** describes the correlation between 22 types of immune cells and analyzes the relationship between different immune cells and survival **(S2 and S3 Tables)**. Interestingly, we found that activated mast cells are the most significant factor negatively correlated with survival.

#### 1.2 TME model classification

The unsupervised hierarchical clustering method was used to identify the TMEcluster, and the classification results of TMEcluster are shown in **S4 Table**. First, according to the elbow method **(Fig 1C)** and gap statics **(Fig 1D)** to determine the optimal classification K value, as shown in **Fig 1C**, in the elbow method, when K = 3, the decline slows down. Elbow method clustering **(Fig 1C)** and gap statistics clustering **(Fig 1D)** are two methods for determining the number of clusters. The elbow method was to find the elbow (that is, the point where the sum of square errors within the group decreases most rapidly), we could clearly see that the elbow point is at K = 3 **(Fig 1C)**.The gap statistic determined the best classification by finding the point with the largest gap, which is K = 7 **(Fig 1D)**. Combining the two methods, according to experience, it was obviously inappropriate to divide into 7 categories, finally we chose to divide it into3 categories. And three main TME cell infiltration subtypes revealed by the data showed significant difference in survival (*log-rank test*, *P< 0*.*001*, **Fig 1E**). Second, we combined the ConsensusClusterPlus function to iterate 1000 times (K = 1: 10) to stabilize the classification and obtained the classification of the sample. It was found that when K = 3, the TMEcluster classification is better **(S1A–S1D Fig)**. Last, mapping the classification of TMEcluster to the immune cell proportion map, we could see that there are obvious difference between different TMEcluster **(Fig 1F and 1G)**.

#### 1.3 Calculate TMEscore and analyze whether TMEscore is related to survival

**Fig 2:**
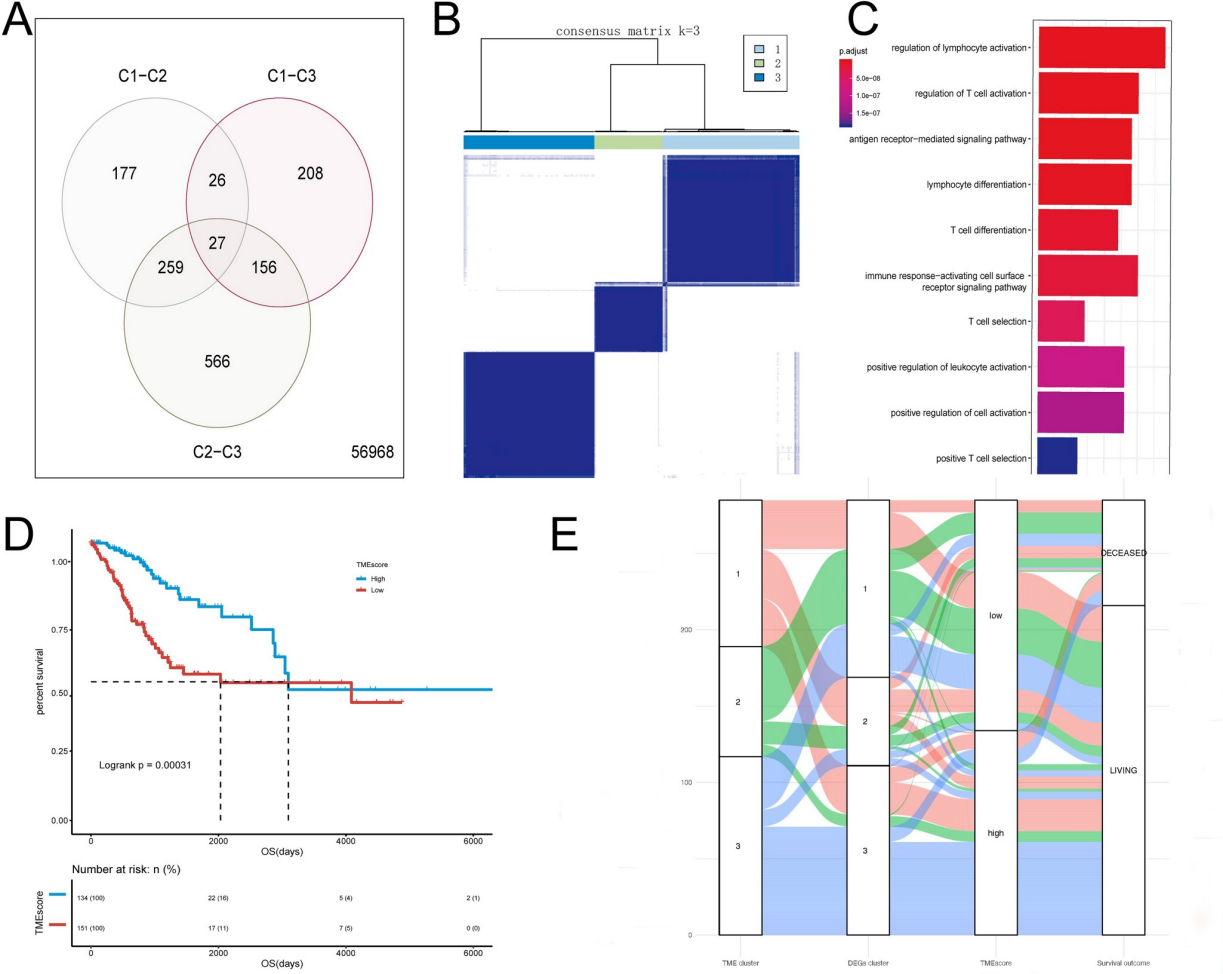
Calculate TMEscore and analyze whether TMEscore is related to survival. (A). Venn diagram (the difference Analysis among 3 different TMEcluster): obtaine a total of 1,419 differentially expressed genes (DEGs). (B). heatmap of consensus matrix (consensus matrix K = 3).: perform unsupervised clustering based on DEGs (n = 1,419) to divide the samples into 3 categories. (C). Functional enrichment analysis of signature genes (n = 84) (D). Kaplan–Meier curves for high (n = 134) and low (n = 151) TMEscore patient groups in the TCGA-CESC cohort. Log-rank test, *P*< 0.001. (E). Alluvial diagram of TMEcluster in groups with different DEGs clusters, TMEscore group and survival outcomes showing difference among patients by cluster.

According to the above TME classification (K = 3), R’s limma package was used to screen different classes of differentially expressed genes (*adj*.*P values* <0.05, | log2FC |> log2 (1.5)) for differential gene analysis. As shown in Venn diagram **(Fig 2A)**, we obtained a total of 1,419 differentially expressed genes **(S5 Table)**. Unsupervised clustering based on the differentially expressed genes was used to divide the sample into three categories **(Fig 2B, S6 Table)**. Next, We used the random forest algorithm to de-redundant differentially expressed genes and selected the signature genes (N = 82) that are most relevant to classification **(S7 Table)**. Using R’s ClusterProfiler package to perform functional enrichment analysis of these 82 genes, it could be seen that these genes are significantly enriched in immune-related pathways such as regulation of lymphocyte activation, regulation of T cell activation **(Fig 2C, S8 Table)**. Therefore, we used Cox regression model to judge the relationship between signature genes (N = 82) and the survival of the samples, then divided signature genes (N = 82) into two categories according to the coefficient value of the genes. Finally we used the TMEscore calculation formula to score TMEscore for all samples. The maximum selection test was used to find the best cut-off point (-0.0249498902040117) to divide the samples into TMEscore high and TMEscore low **(S9 Table)**. The Kaplan–Meier curves of survival analysis for two TMEscore groups **(Fig 2D, S10 Table)** showed that, the TMEscore high group has a good prognosis, while the TMEscore low group has a poor prognosis *(log-rank test*, *P< 0*.*001*), which indicates that clustering the sample based on the immune cell component combined with TMEscore calculation can well characterize the prognosis of samples. **Fig 2E** visualized the alluvial diagram of TMEcluster and TMEscore group.

#### 1.4 TCGA-CESC HPV infection samples, GSE52903 and GSE44001 for validation and evaluation of TMEscore model

**Fig 3:**
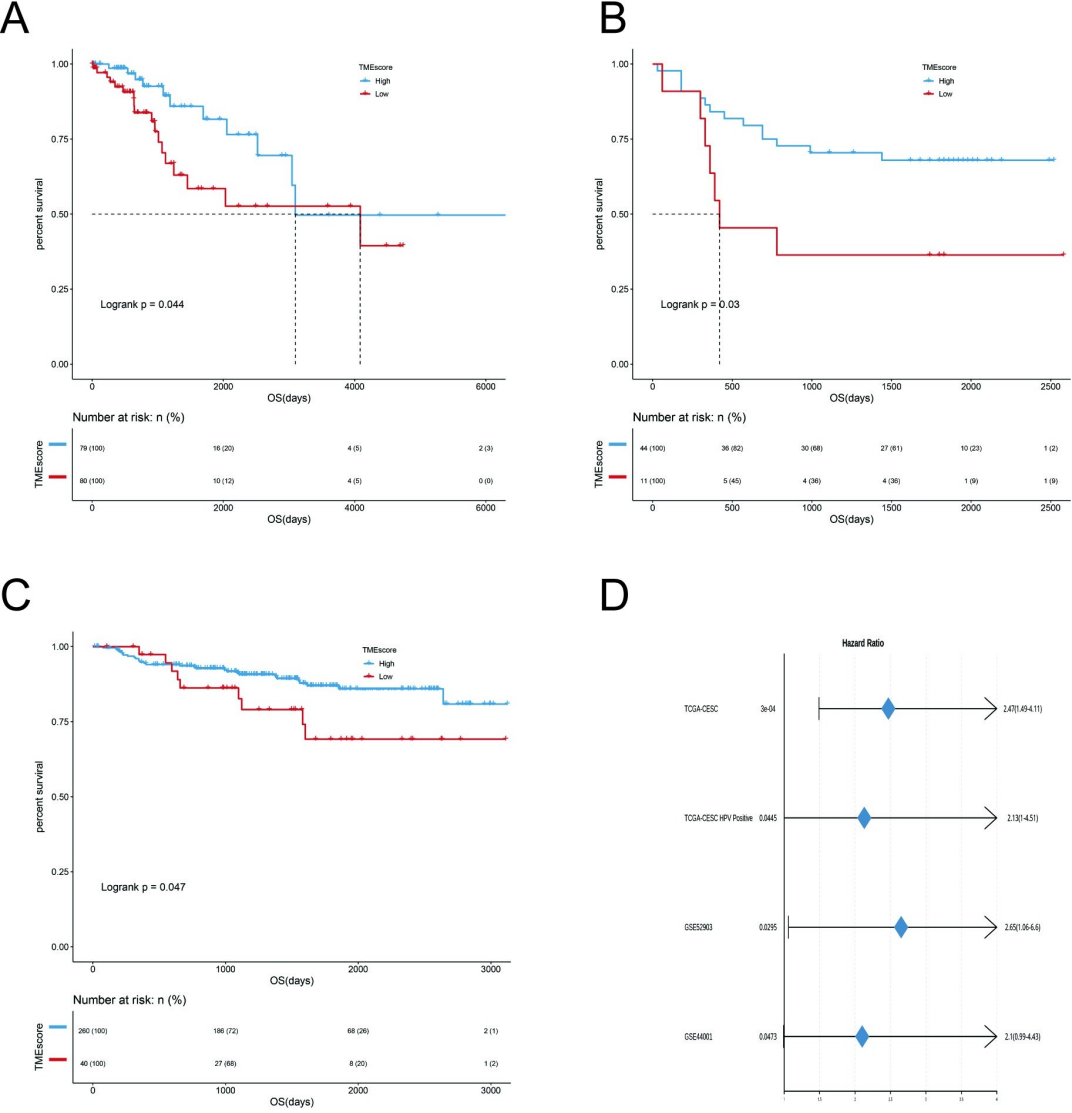
TCGA-CESC HPV infection samples, GSE52903 and GSE44001 for validation and evaluation of TMEscore model. (A). Survival analysis of TCGA-CESC HPV infection samples: Kaplan–Meier curves (OS) for high (n = 79) and low (n = 80) TMEscore patient groups in the TCGA-CESC HPV infection cohort. Log-rank test, *P*< 0.05. (B). Survival analysis of GSE52903: Kaplan–Meier curves (OS) for high (n = 44) and low (n = 11) TMEscore patient groups in the GSE52903 cohort. Log-rank test, *P*< 0.05. (C). Survival analysis of GSE44001: Kaplan–Meier curves (DFS) for high (n = 260) and low (n = 40) TMEscore patient groups in the GSE44001 cohort. Log-rank test, *P*< 0.05. (D). Forest maps for Survival analysis of four samples: TCGA-CESC (*HR* = 2.47,95% *CI* = 1.49–4.11), TCGA-CESC HPV infection samples (*HR* = 2.13,95% *CI* = 1–4.51), GSE52903 (*HR* = 2.65, 95% *CI* = 1.06–6.6), GSE44001 (*HR* = 2.1, 95% *CI* = 0.99–4.43).

According to the previously obtained TMEscore model, TCGA-CESC HPV infection samples(n = 159), GSE52903 (n = 55), and GSE44001(n = 300)were used to evaluate the model effect. As the **Fig 3A–3D** shows, the obtained TMEscore can well characterize the prognosis of the samples. The TMEscore model has relatively good results in TCGA-CESC (*HR = 2*.*47*,*95%CI = 1*.*49–4*.*11)*, TCGA-CESC HPV infection samples (*HR = 2*.*13*,*95%CI = 1–4*.*51)*, GSE52903 (*HR = 2*.*65*,*95%CI = 1*.*06–6*.*6*), GSE44001 (*HR = 2*.*1*,*95%CI = 0*.*99–4*.*43*), indicating that TMEscore model is a very good indicator for assessing prognosis.

### 2. TMEscore significantly correlates with cervical cancer prognosis

Samples with survival time of less than 30 days were further removed, thus 265 cervical cancer samples were finally retained for subsequent analysis. The TMEscore distribution of the retained 265 cervical cancer samples was shown in **S2A Fig. S2B Fig** showed survival analysis of the retained 265 cervical cancer samples. As shown in Kaplan–Meier curves (OS) for high (n = 126) and low (n = 139) TMEscore patient groups, median survival of the high score group is longer than low score group (3097 days vs 2032 days), it is statistically different as indicated by the log-rank test *P* = 0.00025. Then we performed correlation analysis on TMEscore and American Joint Committee on Cancer (AJCC) clinical stage, but the box-plot (**S2C Fig)** showed that the overall correlation between TMEscore and each stage was not statistically significant (*Kruskal-Walis test*, *P = 0*.*45*).

### 3. Comparison of gene expression profile with TMEscores in CC

**Fig 4:**
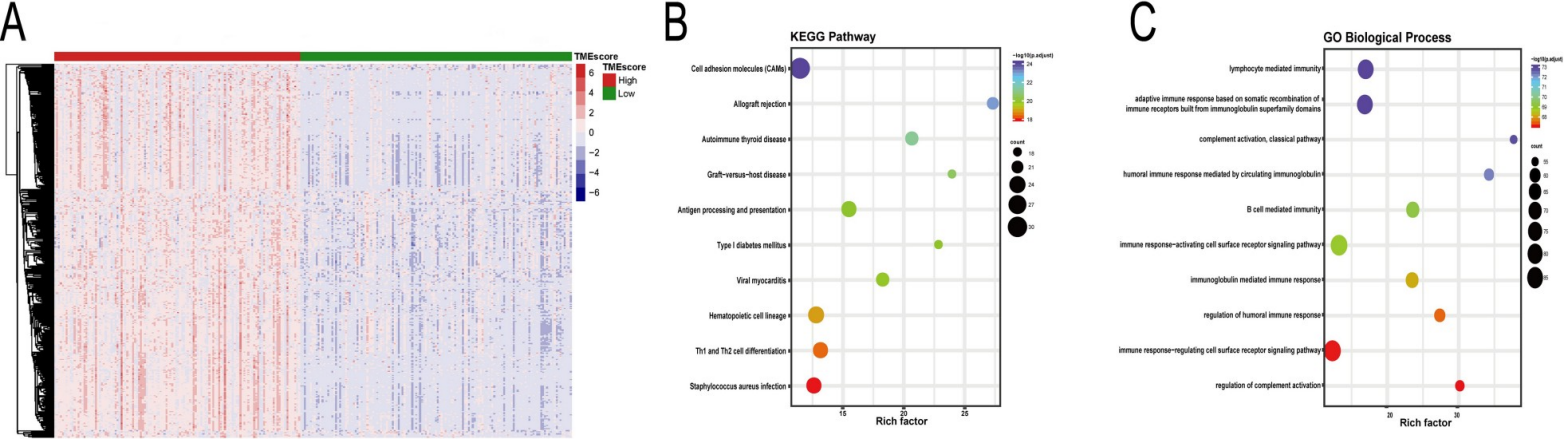
Comparison of gene expression profile with TEMscores in cervical cancer. (A) Heatmap of the DEGs of TMEscores of high score vs low score. (*adj*.*P<0*.*05*, *|log (Fold Change)|>1*). (B). KEGG enrichment of DEGs (C). GO Biological Process enrichment of DEGs.

To reveal the correlation between gene expression and TMEscores, we compared the expression profile data of CC patients in the TCGA database and identified 352 differentially expressed genes (DEGs, **S11 Table**) by grouping according to TMEscores (126 cases/139 cases), which including 351 up-regulated genes and 1 down-regulated genes as the key genes for subsequent analysis. **(Fig 4A)**. Next, in order to outline the potential functions of these DEGs above, we performed GO and KEGG enrichment analysis on DEGs respectively. The enrichment analysis results showed that DEGs significantly enrich in immune-related functions. **(S12 and S13 Tables and Fig 4B and 4C: choose TOP10 for demonstration)**.

### 4. Survival analysis for DEGs

**Fig 5:**
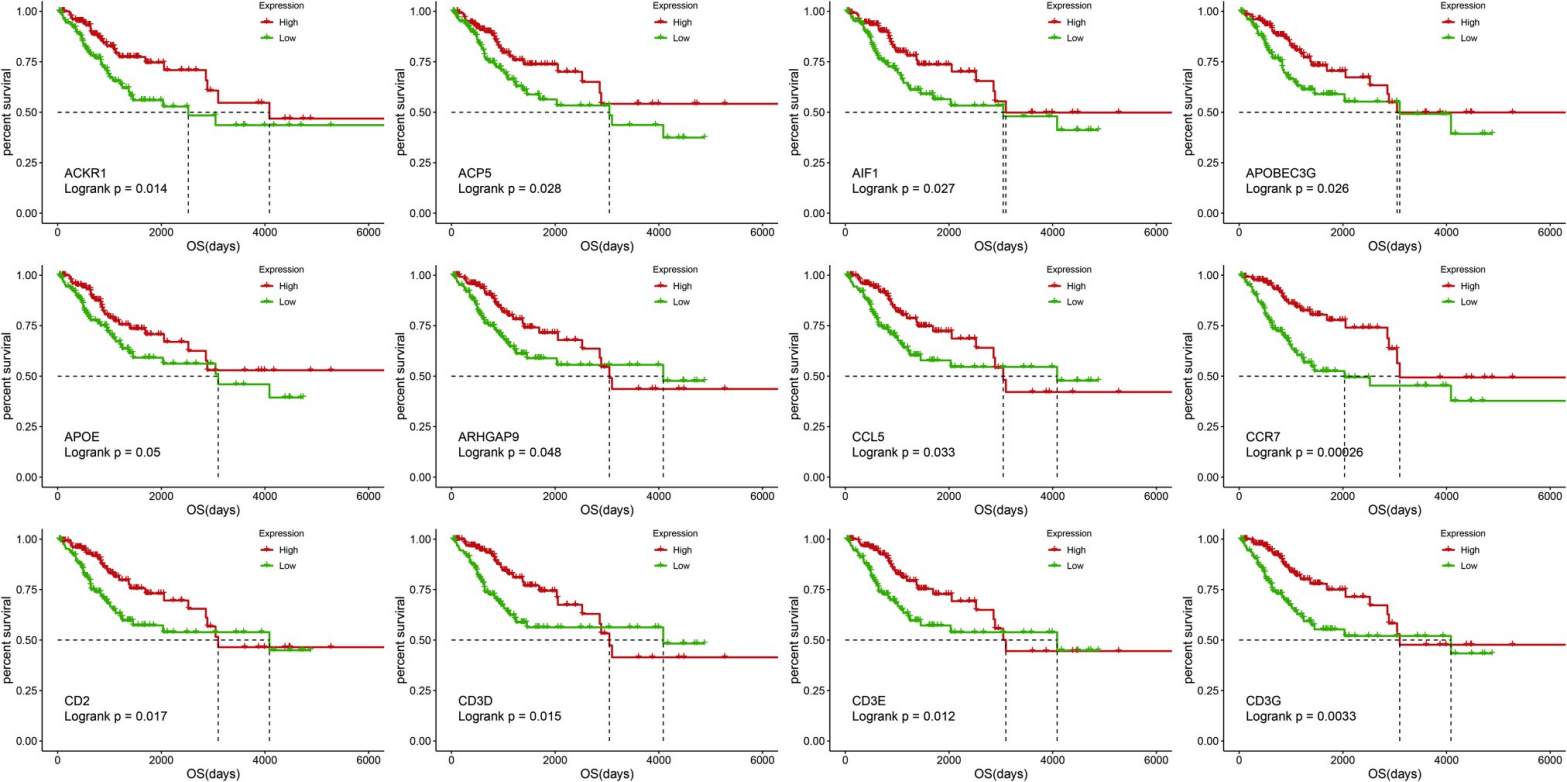
Kaplan-Meier survival curves of some prognosis genes. Red line represents high gene expression group and green line represents low gene expression group. *P*<0.05 in Log-rank test. overall survival (OS) in days.

In order to screen out genes related to the prognosis of CC, we divided these 352 DEGs into high and low expression groups according to their median expression and performed survival analysis **(S14 and S15 Tables)**. Among the 352 DEGs, a total of 86 survival-related prognosis genes **(S16 Table)** were excavated (*log-rank test*, *P<0*.*05*). Selected prognosis gene survival curves are shown in **Fig 5**.

### 5. Functional enrichment analysis of prognosis genes

**Fig 6:**
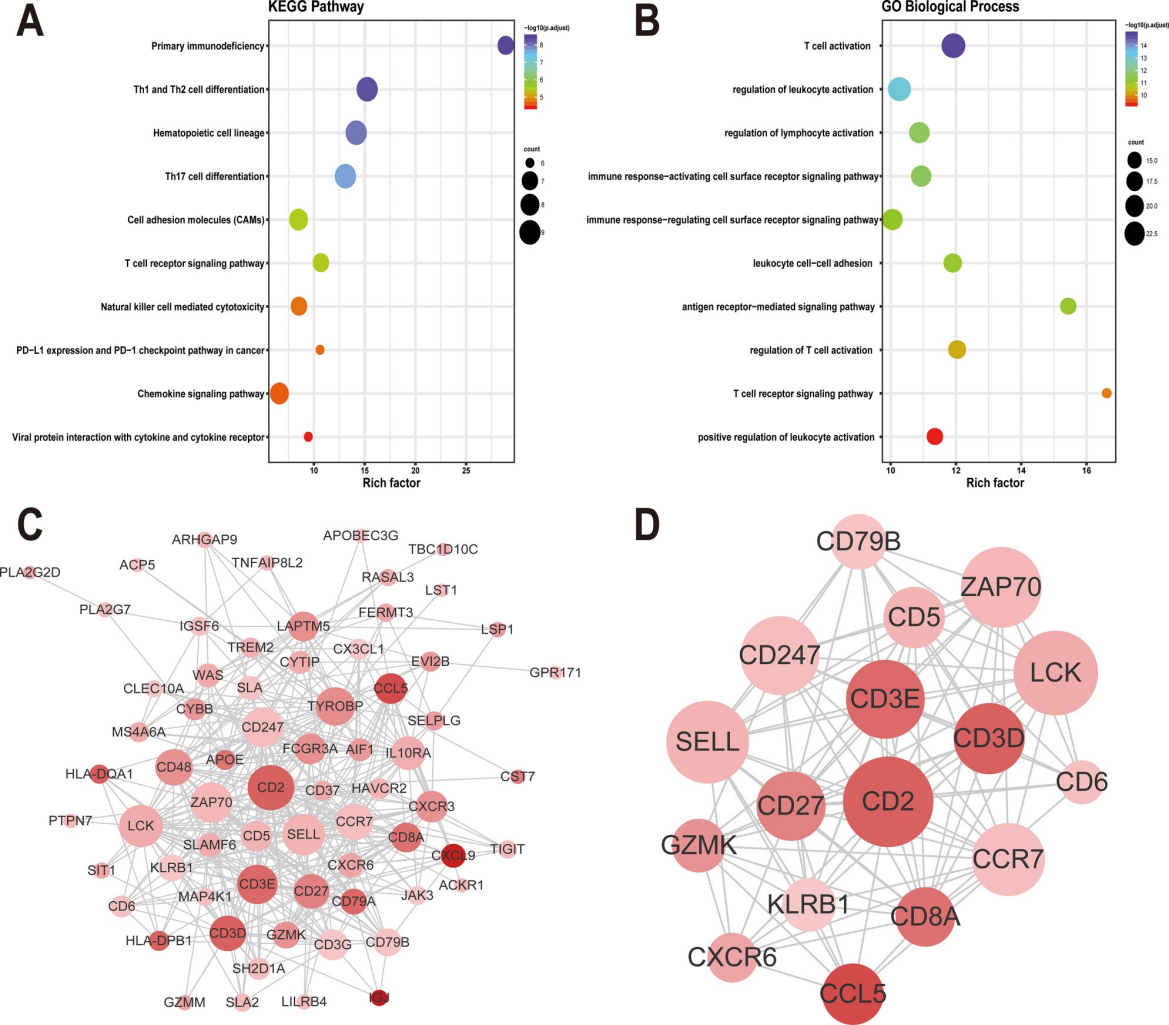
Functional enrichment analysis and PPI network of prognosis genes. (A) GO Biological Process enrichment of prognosis genes. (B). KEGG enrichment of prognosis genes. (C). PPI network of prognosis genes. (D). MCODE module mining: The node size represents degree, and the node color from light to dark represents log (FC) value from small to large.

KEGG pathway and GO enrichment analysis were performed on 86 prognosis genes. The enrichment analysis results still show significant enrichment in immune-related functions, such as Primary immunodeficiency (hsa05340), Th1 and Th2 cell differentiation (hsa04658), Th17 cell differentiation (hsa04659), T cell activation (GO:0042110), regulation of leukocyte activation (GO:0002694), antigen receptor-mediated signaling pathway (GO:0050851), etc. **(Fig 6A, 6B and S17 and S18 Tables)**.

### 6. PPI network construction and module mining for prognosis genes

To better understand the interactions between the identified prognostic genes, we used the STRING database to obtain a protein-protein interaction (PPI) network that includes 68 nodes and 400 edges **(Fig 6C)**. Next, in order to further mine the information in the network, we used the MCODE plug-in to further mine the interacting modules. In the end, only 1 module was mined, including 17 nodes and 104 edges **(Fig 6D)**. As shown in **Fig 6D**, the center of the module is mainly occupied by key immune response genes such as CD3D, CD3E, CD8A, and CD27, etc. These genes were defined as module genes.

## Discussion

The overall analysis process of this study is shown in **S3 Fig**. First, we performed CIBERSORT analysis using RNA-seq data from TCGA-CESC cervical cancer samples to obtain the TME infiltration pattern of 285 cervical cancer patients **(Fig 1**). Second, we performed the principal component analysis algorithm to build a TMEscore model **(Fig 2)**, which is divided into two groups: TMEscore high and TMEscore low. To prove that TMEscore is a better prognostic marker, the alluvial diagram **(Fig 2E)** of TMEcluster in groups with different DEGs clusters, TMEscore group and survival outcomes showing difference among patients by cluster. It is worth noting that in this study, we performed consistent clustering based on the results of CIBERSORT, and the results showed that the optimal number of clusters was 3 (TMEcluster1, TMEcluster2, TMEcluster3), then we did a survival analysis based on these three categories, and the *P* = 0.0087 **(Fig 1E)**. At the same time, we used TMEscore’s classification results (TMEscore low, TMEscore high) for survival analysis, and the *P* = 0.00031 **(Fig 2D)**. The comparison shows that although TMEscore is based on CIBERSORT, TMEscore is better than the immune cell infiltration based classification. Next, the TMEscore was validated for TCGA-CESC HPV infection samples, GSE52903, GSE44001 **(Fig 3)**. In **Fig 3C**, the Kaplan Meier curve (DFS) difference between the high (n = 260) and low (n = 40) TMEscore patient groups in the GSE44001 cohort is statistically significant. However, in **Fig 3D** (Forest maps for Survival analysis of four samples), the GSE44001 sample (*HR = 2*.*1*, *95% CI = 0*.*99–4*.*43*) did not show statistical significance. We think that this result may be caused by the imbalance of the grouped samples and the relatively small overall sample. Because CC is significantly associated with infectious agents, most notably human papillomavirus (HPV) [14], However, due to database limitations, we have not used the corresponding data to verify that the TMEscore model is a prognostic biomarker for immune checkpoint inhibitor responses, which is deficiencies in the study.

Next, we attempted to identify TME related genes that contribute to CC overall survival (OS) in the TCGA database. In particular, by comparing global gene expression of the two groups of TMEscore high and TMEscore low, we extracted 352 differentially expressed genes and found that many of them are related to immune-related functions, as shown by GO **(Fig 4B)** and KEGG **(Fig 4C)** analysis. Then we performed survival analysis of these 352 genes and determined that 86 genes were associated with prognosis for patients with CC. The analysis of the 86 prognosis genes GO **(Fig 6A)** and KEGG **(Fig 6B)** also showed significant enrichment in immune-related functions. Interestingly, we identified some pathways that are not directly related to immune-related pathways in **S8 Table,** such as: positive regulation of cell activation (GO:0050867); positive regulation of cell adhesion (GO:0045785); cellular calcium ion homeostasis (GO:0006874); purinergic receptor signaling pathway (GO:0035587); calcium-mediated signaling (GO:0019722); positive regulation of JNK cascade (GO:0046330), etc. Finally, we constructed and finally mined a PPI network of prognosis genes **(Fig 6D)**, all of which are related to the immune response. Highly relevant nodes in the module include CD3D, CD3E, and CD8A, which are mainly expressed in CD8^+^T lymphocyte lines. The limitation of this study is that the results only obtained only through bioinformatics analysis. We have not further conducted genetic and experimental studies with larger sample sizes to confirm the TMEscore model.

In summary, we first proposed the establishment of a TMEscore model to predict the prognosis of CC. In this study, we used the TME infiltration pattern of TCGA-CESC CC patients and used the principal component analysis algorithm to obtain a TMEscore model. The TMEscore was validated as a powerful prognostic biomarker by other data. By comparing the global gene expression and survival analysis of TMEscore high/low groups, we obtained a list of prognostic genes (N = 86, **S16 Table**), which may help to describe the prognosis of CC patients. Some previously ignored genes may become additional biomarkers for CC. In addition, further research into prognostic genes may lead to new insights into the potential link between TME and CC prognosis.

## Supporting information

S1 Fig TME model classification. Consensus matrixes of TCGA-CESC cohort for each k (k = 2–5), displaying the clustering stability using 1000 iterations of hierarchical clustering. (A). heatmap of consensus matrix(K = 2). (B). heatmap of consensus matrix (K = 3). (C). heatmap of consensus matrix(K = 4) (D). heatmap of consensus matrix (K = 5) vertical axis represents samples, horizontal axis represents the classification of consensus matrix. The more neat the classification, the better the classification effect. (E) Consensus Cumulative Distribution Function (CDF) Plot: vertical axis represents the consensus index, and horizontal axis represents the probability.
(TIF)

S2 Fig TMEscore significantly correlates with cervical cancer prognosis. (A). Score distribution of the retained 265 cervical cancer samples with survival time of more than 30 days. (B). Survival analysis of the retained 265 cervical cancer samples. As shown in Kaplan–Meier curves (OS) for high (n = 126) and low (n = 139) TMEscore patient groups, median survival of the high score group is longer than low score group (3097 days *vs* 2032 days), it is statistically different as indicated by the log-rank test *P* = 0.00025. (C). Distribution of TEMscores of CC stage (AJCC). Box-plot shows the association between TMEscore and cervical cancer stage, but it is not statistically significant (*Kruskal-Walis test*, *P = 0*.*45*).
(TIF)

S3 Fig Overall project flow. (TIF)

S1 Table Sample information of cervical cancer patients. (XLSX)

S2 Table Correlation between 22 immune cells. (XLSX)

S3 Table The relationship between different immune cells and survival. (XLSX)

S4 Table TMEcluster grouping results. (XLSX)

S5 Table 1419 differentially expressed genes. (XLSX)

S6 Table Unsupervised clustering of differentially expressed genes. (XLSX)

S7 Table The signature genes (N = 82) that are most relevant to classification. (XLSX)

S8 Table Enrichment analysis results of signature genes (N = 82). (XLSX)

S9 Table TMEscore classification results. (XLSX)

S10 Table TMEscore and grouping information of samples used for subsequent analysis. (XLSX)

S11 Table List of DEGs (n = 352). (XLSX)

S12 Table Results of KEGG enrichment analysis of DEGs (n = 352). (XLSX)

S13 Table Results of GO enrichment analysis of DEGs (n = 352). (XLSX)

S14 Table DEGs expression summary table about survival information. (XLSX)

S15 Table Survival analysis P value of DEGs. (XLSX)

S16 Table List of prognosis genes (n = 86). (XLSX)

S17 Table Results of KEGG enrichment analysis of prognosis genes (n = 86). (XLSX)

S18 Table Results of GO enrichment analysis of prognosis genes (n = 86). (XLSX)

S1 Data (CSV)

S2 Data (TXT)

S3 Data (TXT)

S1 File (PDF)

S2 File (PDF)

## Abbreviations

CC: Cervical cancer
TME: Tumor microenvironment
TCGA: the cancer genome atlas
GEO: Gene Expression Omnibus
TMEscore: TME signature score
TIC: tumor-infiltrating immune cell
HPV: human papillary virus
CIBERSORT: Cell type Identification By Estimating Relative Subsets Of RNA Transcripts
FDR: False discovery rate
GO: Gene ontology
BP: biological processes
KEGG: Kyoto encyclopedia of genes and genomes
DEGs: Differentially expressed genes
OS: overall survival
DFS: progression-free survival the
PPI: protein-protein interaction
CD8: cluster of differentiation 8
