# Supplementary figures and images for: Tumor microenvironment characterization in cervical cancer identifies prognostic relevant gene signatures
Source: PLoS One. 2021 Apr 26;16(4):e0249374. doi: 10.1371/journal.pone.0249374 (PMC8075229; doi:10.1371/journal.pone.0249374)

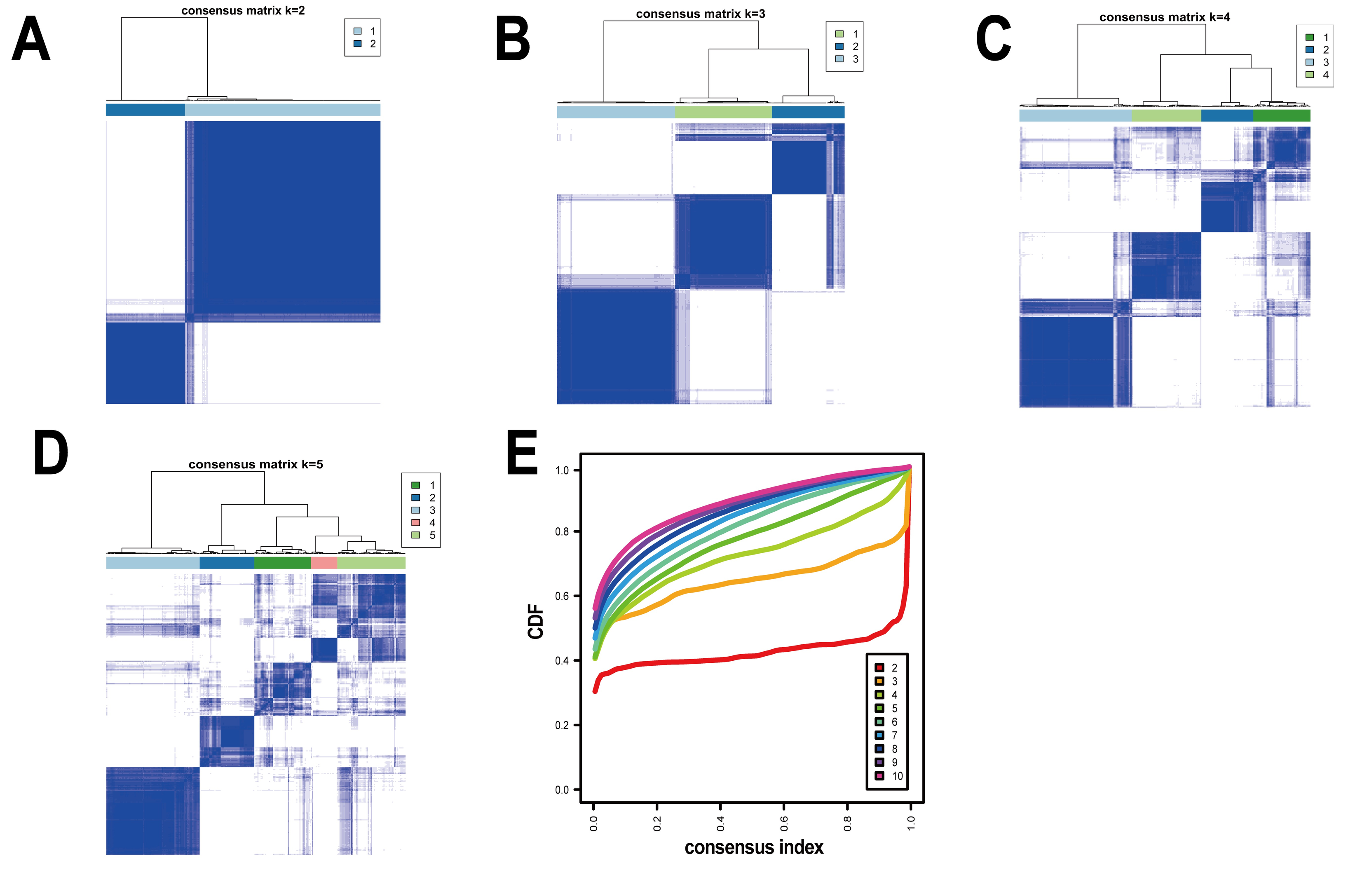

Supplement: S1 Fig — Consensus matrixes of TCGA-CESC cohort for each k (k = 2–5), displaying the clustering stability using 1000 iterations of hierarchical clustering. (A). heatmap of consensus matrix(K = 2). (B). heatmap of consensus matrix (K = 3). (C). heatmap of consensus matrix(K = 4) (D). heatmap of consensus matrix (K = 5) vertical axis represents samples, horizontal axis represents the classification of consensus matrix. The more neat the classification, the better the classification effect. (E) Consensus Cumulative Distribution Function (CDF) Plot: vertical axis represents the consensus index, and horizontal axis represents the probability. (TIF) [file pone.0249374.s001.tif]

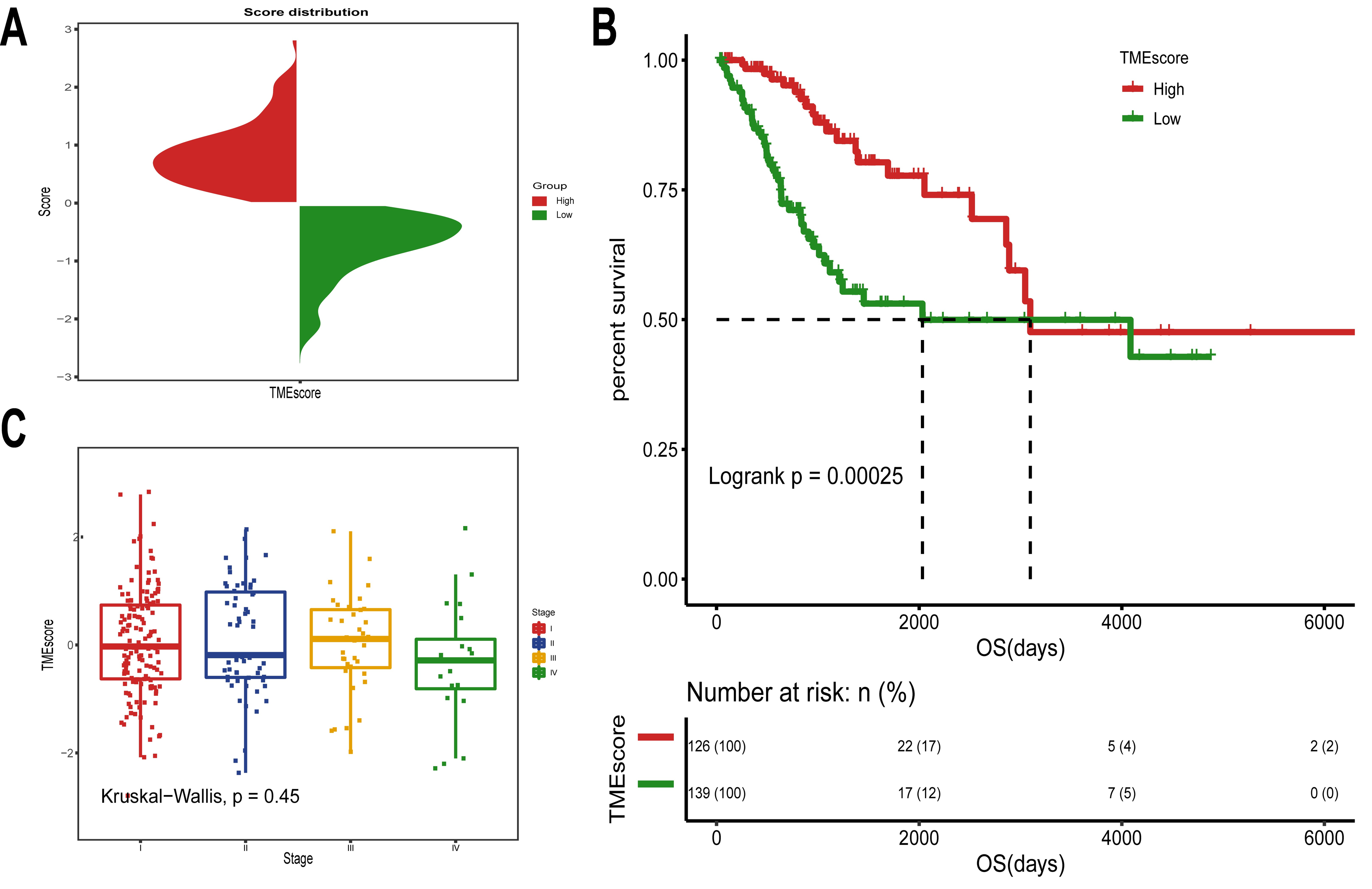

Supplement: S2 Fig — (A). Score distribution of the retained 265 cervical cancer samples with survival time of more than 30 days. (B). Survival analysis of the retained 265 cervical cancer samples. As shown in Kaplan–Meier curves (OS) for high (n = 126) and low (n = 139) TMEscore patient groups, median survival of the high score group is longer than low score group (3097 days vs 2032 days), it is statistically different as indicated by the log-rank test P = 0.00025. (C). Distribution of TEMscores of CC stage (AJCC). Box-plot shows the association between TMEscore and cervical cancer stage, but it is not statistically significant (Kruskal-Walis test, P = 0.45). (TIF) [file pone.0249374.s002.tif]

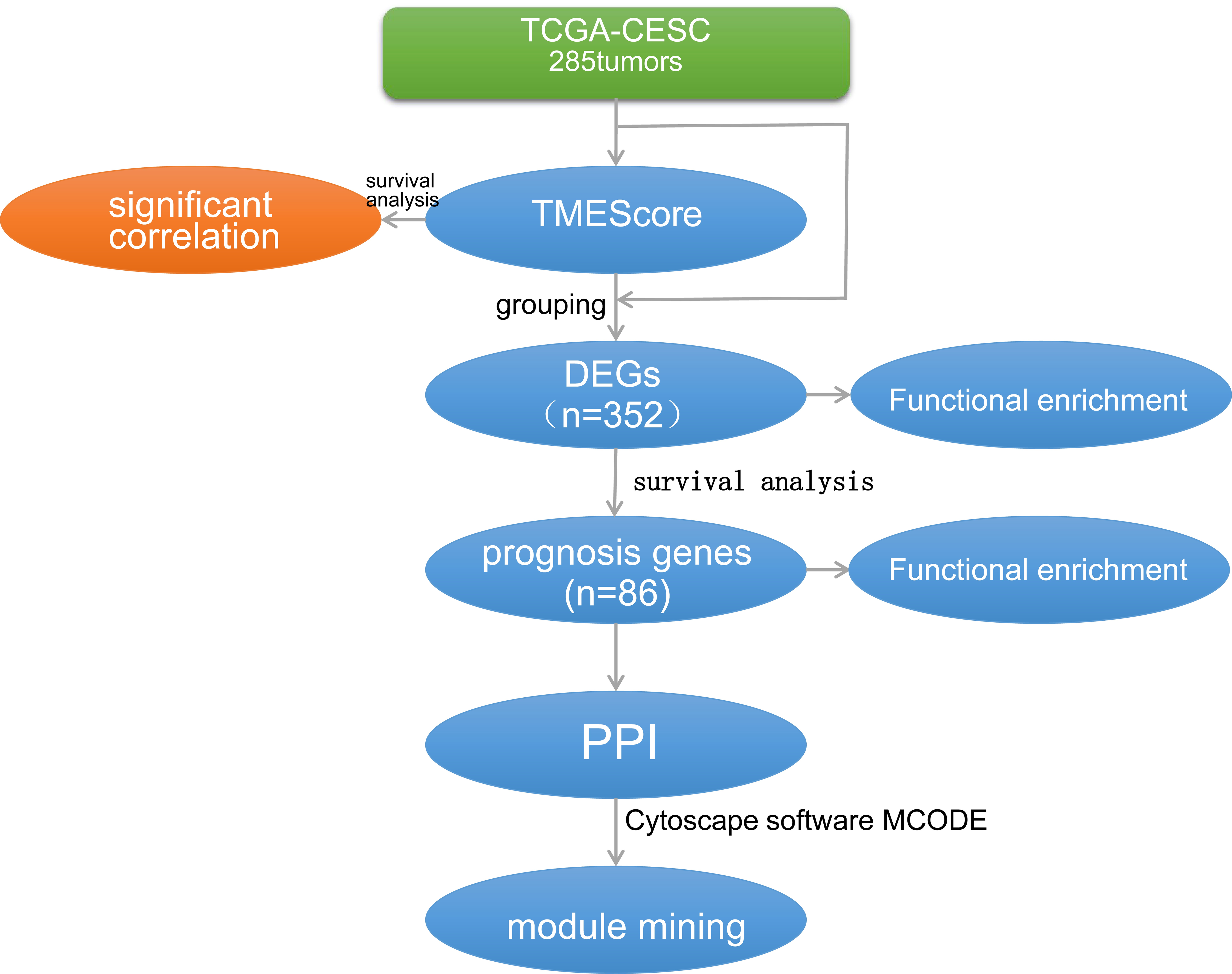

Supplement: S3 Fig — (TIF) [file pone.0249374.s003.tif]

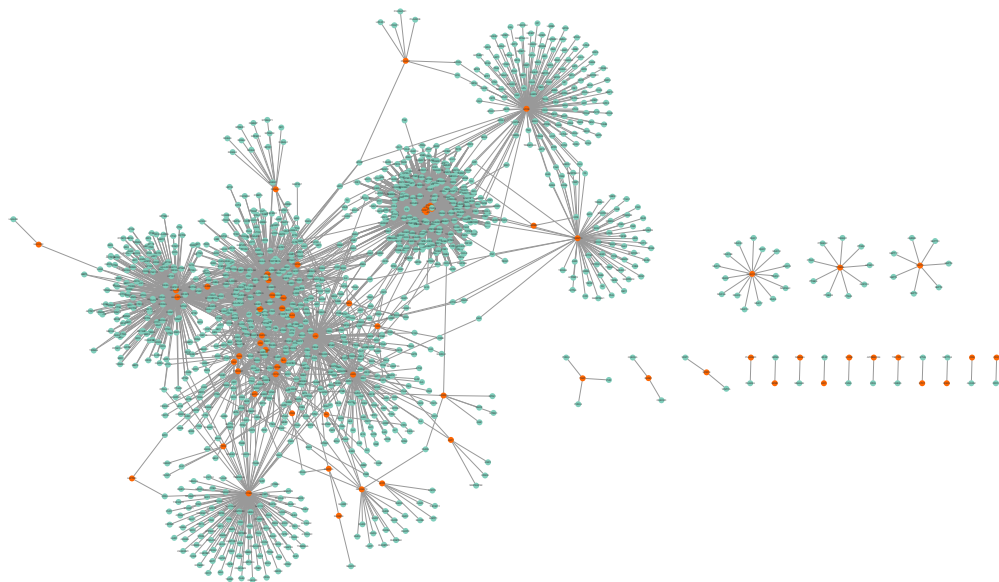

Supplement: S1 File — (PDF) [file pone.0249374.s025.pdf]
