# Supplementary material for: Tumor microenvironment characterization in cervical cancer identifies prognostic relevant gene signatures
Source: PLoS One. 2021 Apr 26;16(4):e0249374. doi: 10.1371/journal.pone.0249374 (PMC8075229; doi:10.1371/journal.pone.0249374)
Supplement: S2 File — (PDF) [file pone.0249374.s026.pdf]

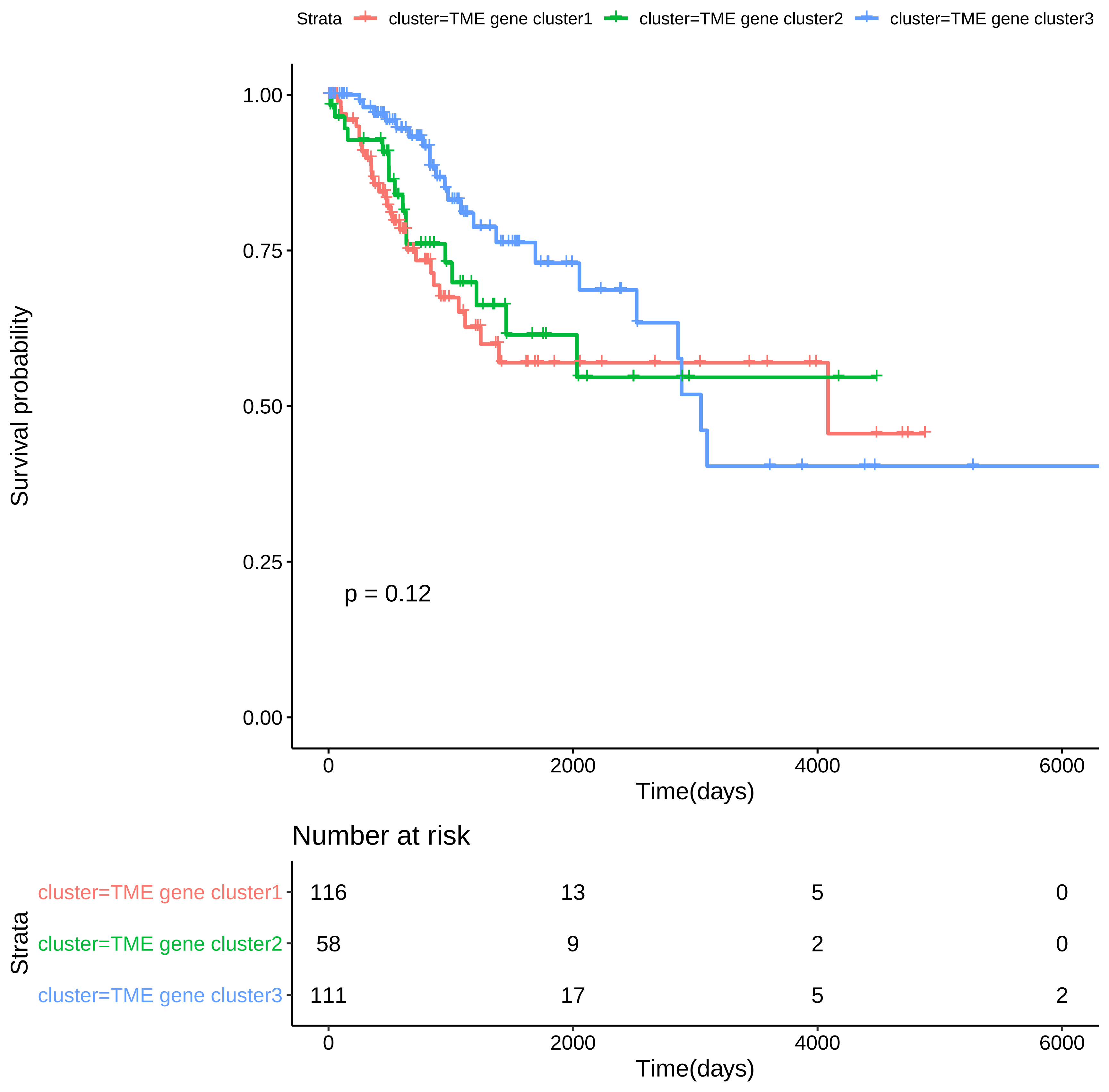

Strata
cluster=TME gene cluster1
cluster=TME gene cluster2
cluster=TME gene cluster3
Survival probability
1.00
0.75
0.50
0.25
0.00
p = 0.12
0
2000
4000
6000
Time(days)
Number at risk
Strata
cluster=TME gene cluster1
116
13
5
0
cluster=TME gene cluster2
58
9
2
0
cluster=TME gene cluster3
111
17
5
2
0
2000
4000
6000
Time(days)
